# Epidemiology of Syphilis in regional blood transfusion centres in Burkina Faso, West Africa

**DOI:** 10.11604/pamj.2013.16.69.2767

**Published:** 2013-10-28

**Authors:** Cyrille Bisseye, Mahamoudou Sanou, Bolni Marius Nagalo, Alice Kiba, Tegwindé Rebeca Compaoré, Issoufou Tao, Jacques Simpore

**Affiliations:** 1 Pietro Annigoni Biomolecular Research Centre (CERBA)/ Labiogene, University of Ouagadougou, Burkina Faso; 2 Laboratory of molecular and cell Biology, University of Science and Technique of Masuku (USTM), Franceville, Gabon; 3 National Transfusion centre, Ouagadougou, Burkina Faso

**Keywords:** Syphilis, HIV, blood donor, seroprevalence, Burkina Faso

## Abstract

**Introduction:**

Syphilis remains a major public health problem in sub-Saharan Africa, including Burkina Faso. However, few published data are available on the prevalence of syphilis in the general population. This study had two main objectives: to determine the seroprevalence of syphilis in a cohort of 37,210 first time blood donors and to study socio-demographic factors associated with the risk of infection by *Treponema pallidum*.

**Methods:**

Antibodies to *Treponema pallidum* were screened for, by using Reagin Rapid Test (RPR) and their presence was confirmed by *treponema pallidum* haemagglutination test (TPHA).

**Results:**

The overall seroprevalence of syphilis was 1.5% among first time blood donors and was significantly different between centers (p <0.001). The infection was significantly higher in men than women among blood donors in Ouagadougou and Fada N′gourma (P = 0.001 and P = 0.034). The overall seroprevalence of syphilis among blood donors was not associated with either age group or HIV status. In contrast, a significantly high seroprevalence of syphilis was observed in blood donors with HBsAg (P = 0.014) and anti-HCV (P = 0.007) positive.

**Conclusion:**

Our report shows a low seroprevalence of syphilis in the representative sample of the population of Burkina Faso. The seroprevalence of syphilis remains unequally distributed between urban and rural areas and was not associated with HIV infection.

## Introduction

According to the World Health Organization (WHO), each year about 340 million new infections are due to sexually transmitted diseases such as chlamydia, gonorrhea, syphilis and Trichomonas [1]. Syphilis remains a major public health problem in sub-Saharan Africa, including Burkina Faso. It is diagnosed routinely in all blood donors using non-treponemal and treponemal tests such as Rapid Plasma Reagin test (RPR) and *T. pallidum* haemagglutination Test (TPHA) [2, 3]. Screening for syphilis in Burkina Faso is performed for pregnant women prenatal medical examination of pregnant women, medical prescription in case of clinical suspicion, for the United States of America visa's applicants and for medical screening among new recruits of the army. In Burkina Faso, few published data are available on the prevalence of syphilis in the population. Previous studies have reported a regional variation in the prevalence of syphilis among pregnant women [4, 5] and blood donors[6, 7]; however, most of these studies had several limitations. In fact, they involved a small sample size and the sociodemographic factors associated with the risk of syphilis infection were not systematically studied. Furthermore, the relationship between syphilis and other viruses such as human immunodeficiency virus (HIV), hepatitis B and C viruses (HBV and HCV) were not always considered.

This study has two main objectives: to determine the seroprevalence of syphilis in a cohort of 37,210 first time blood donors recruited in the regional blood transfusion centres of Ouagadougou, Bobo-Dioulasso, Fada N′gourma and Koudougou and to study the socio-demographic factors associated with the risk of infection by *Treponema pallidum* in the population.

## Methods

### Donors recruitment

**Figure 1:**
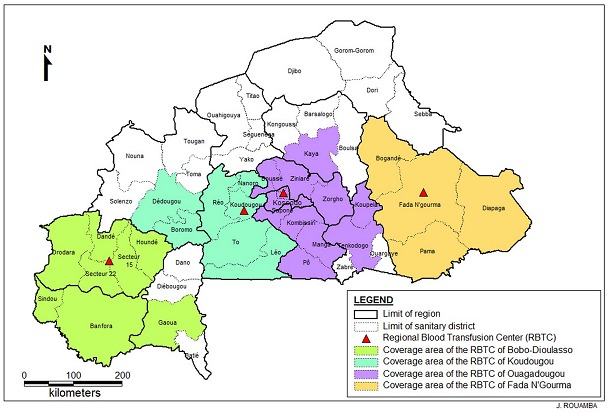
Regional blood transfusion centres and area of coverage in Burkina Faso in 2010

A retrospective analysis of blood donors' data from January to December 2010 was conducted in 4 regional blood transfusion centres in Burkina Faso: Ouagadougou (Central region), Bobo-Dioulasso (High-Basins), Koudougou (Central West region) and Fada N'gourma (Eastern Region). The four blood transfusion centres cover the needs in blood products of the surrounding group of provinces as shown in Figure 1. Voluntary donors were all healthy subjects, selected after responding to a panel of questions comprising a medical background ; and individuals aged 17-64 years with a weight >50 kg, were included for blood donation. All donors answered questions intending to exclude recipients of previous blood transfusion, individuals having experienced jaundice or signs of hepatitis, pregnant women and people having experienced a high-risk sexual behaviour within 2 weeks preceding the intended donation. The socio-demographic characteristics of selected donors were recorded in a database, and venous blood was collected in blood banking bags following standard procedures. In each centre, blood collection was conducted as previously described [8]

Ethical considerations: This study was approved by the CERBA/Saint Camille Ethics Committee. However, because of the retrospective nature of the study, informed consent was not obtained from the study subjects.

Serological analysis: Antibodies to Treponema pallidum were detected using, Rapid Plasma Reagin test (RPR; Cypress Diagnostics, Langdorp, Belgium) and their presence confirmed by *Treponema pallidum* haemagglutination test (TPHA, Cypress Diagnostics, Langdorp, Belgium).

Antibodies to HIV types 1 and 2 were screened for using Vironostika HIV Uni-Form II Ag/Ab (Biomérieux, Boxtel,the Netherlands). All samples reactive for HIV, HBsAg and HCV were re-tested for confirmation using a second enzyme-linked immunosorbent assay (Bio-Rad, Marnes la Coquette, France). A result was considered positive if both the first and second tests were positive.

Statistical analysis: Data were analyzed using Statistical Package for the Social Sciences (SPSS version 17; SPSS Inc. Chicago, IL, USA) and EPI-Info version 6.04 dfr (CDC, Atlanta, GA, USA). Odds ratio was calculated to determine risk factors associated with syphilis. P values below 0.05 were considered statistically significant.

## Results

Of the 37,210 first time blood donors recruited in the four regional blood transfusion centers, 72.5% (95% CI 72.0 to 73.0) were men and 27.5 (95% CI 26.3 to 28.4) were females. The majority of donors belonged to the age group 20-29 years (58.1%, 95% CI 57.4 to 58.8) and were mainly recruited in urban (70.2%) than rural areas (29.8%) (Table 1). The number of blood donors was respectively 16 925 (45.5%), 8859 (23.8%), 6599 (17.7%) and 4827 (13.0%) in blood transfusion centres of Ouagadougou, Bobo- Dioulasso, Fada and N′gourma and Koudougou. The overall seroprevalence of syphilis was 1.5% among first time blood donors and was significantly different between centers (p <0.001), the highest being observed in Koudougou (2.5%) and lowest in Bobo-Dioulasso (0.7%) (Table 2). The overall seroprevalence of syphilis among blood donors was not associated with either age group or HIV status. In contrast, significantly higher seroprevalence of syphilis was observed in blood donors with HBsAg (P = 0.014) and anti-HCV (P = 0.007) positive.

**Table 1:** Sociodemographic characteristics of first-time blood donors in Burkina Faso

Characteristics	Total number	Percentage	95% CI
**Gender**			
Male	26981	72.5	72.0-73.0
Female	10229	27.5	26.3-28.4
**Age groups (years)**			
< 20	8130	21.8	20.9-22.7
20-29	21630	58.1	57.4-58.8
30-39	4792	12.9	12.0-13.9
40-49	2052	5.5	4.5-6.5
≥50	606	1.6	0.6-2.6
**Location of blood donation**			
Urban areas	26123	70.2	69.6-70.8
Rural areas	11087	29.8	28.9-30.7
**Blood groups**			
A	8430	22.7	21.8-23.6
AB	2157	5.8	4.8-6.8
B	10476	28.2	27.3-29.1
O	16147	43.4	42.6-44.2
**Rhesus (RH) Type**			
Positive	32073	86.2	85.8-86.6
Negative	5137	13.8	12.9-14.7

CI = Confidence Interval

**Table 2:** Seroprevalence of syphilis among first-time blood donors in 2010

Characteristics	Total number	Number of syphilis positive	Percentage	P-values
**Blood centres**				
Bobo-Dioulasso	8859	63	0.7	**< 0.001**^a^
Fada N'gourma	6599	99	1.5	
Koudougou	4827	121	2.5	
Ouagadougou	16925	290	1.7	
**Gender**				
Male	26981	458	1.7	**< 0.001**^b^
Female	10229	115	1.1	
**Age groups (years)**				
< 20	8130	114	1.4	0.203^a^
20-29	21630	359	1.7	
30-39	4792	69	1.4	
40-49	2052	23	1.1	
≥50	606	8	1.3	
**Location of blood donation**				
Urban areas	26123	355	1.4	**< 0.001**^b^
Rural areas	11087	218	2.0	
**Co-Infections**				
**HBV**				**0.014^b^**
Positive	4998	97	1.9	
Negative	32212	476	1.5	
**HCV**				
Positive	2513	55	2.2	**0.007**^b^
Negative	34697	518	1.5	
**HIV**				0.662^b^
Positive	802	10	1.2	
Negative	36408	563	1.5	
**Blood groups**				
O	16147	227	1.4	0.068^b^
Non-O	21063	346	1.6	

a Pearson Chi square

b Fischer's Exact test (2-sided); Significant P-values in Bold

The seroprevalence of syphilis was examined in detail by blood transfusion centre as shown in Tables 3 and 4. The infection was significantly higher in men than women among blood donors in Ouagadougou and Fada N′gourma (P = 0.001 and P = 0.034). Syphilis was not associated with any age group in donors of all blood transfusion centers, even though a slightly higher seroprevalence was observed among blood donors in the age group 20-29 years in Ouagadougou (P = 0.052) (Table 3). Regarding the place of blood donation, the seroprevalence of syphilis was significantly higher in rural compared to urban areas of Ouagadougou and Fada N′gourma (P <0.001) (Tables 3 and 4). The association between syphilis and HIV, HBV and HCV was examined in blood donors. There was no association between HIV and syphilis among all donors of all blood transfusion centers. The seroprevalence of syphilis was higher among donors with HBsAg and anti-HCV positive tests in Bobo-Dioulasso (P = 0.003) and Ouagadougou (P <0.001). No socio-demographic factors were associated with syphilis among blood donors in Koudougou (Table 4).

**Table 3:** Socio-demographic characteristics of first-time blood donors at Ouagadougou and Bobo-Dioulasso according to syphilis in 2010

	Ouagadougou				Bobo-Dioulasso			
Characteristics	N Total	Syphilis Positive N (%)	OR (95% CI)	P-values	N Total	Syphilis Positive N (%)	OR (95% CI)	P-values
**Gender**								
Male	11427	223 (2.0)	1.6 (1.2-2.1)	0.001	6903	51 (0.7)	1.2 (0.6-2.4)	0.649
Female	5498	67 (1.2)	1	-	1956	12 (0.6)	1	
**Age groups**								
< 20	3316	54 (1.6)	1.6 (0.8-3.3)	0.147	2371	14 (0.6)	NA	-
20-29	9659	183 (1.9)	1.9 (1.0-3.7)	0.052	5466	45 (0.8)	NA	-
30-39	2510	38 (1.5)	1.5					
(0.7-3.1)	0.237	667	3 (0.4)	NA	-			
40-49	1083	11 (1.0)	1	-	298	1 (0.3)	1	-
≥ 50	357	4 (1.1)	1.1 (0.3-3.8)	0.772	57	0 (0.0)	-	-
**Location of blood donation**								
Urban areas	13265	192 (1.4)	1	-	5670	45 (0.8)	1.4 (0.8-2.5)	0.218
Rural areas	3660	98 (2.7)	1.9 (1.5-2.4)	< 0.001	3189	18 (0.6)	1	-
**HBV**								
AgHBs positive	2057	41 (2.0)	1.2 (0.9-1.7)	0.253	1038	15 (1.4)	2.4 (1.3-4.4)	0.003
AgHBs negative	14868	249 (1.7)	1	-	7821	48 (0.6)	1	-
**HCV**								
Seropositive	879	29 (3.3)	2.1 (1.4-3.1)	< 0.001	532	3 (0.6)	1	-
Seronegative	16046	261 (1.6)	1	-	8327	60 (0.7)	1.3 (0.4-5.1)	0.677
**HIV**								
Seropositive	323	5 (1.5)	1	-	164	0 (0.0)	-	-
Seronegative	16602	285 (1.7)	1.1 (0.4-3.1)	0.817	8695	63 (0.7)	NA	-
**Blood groups**								
O	7373	107 (1.5)	1	-	3766	27 (0.7)	1.0 (0.6-1.7)	0.955
Non-O	9552	183 (1.9)	1.3 (1.0-1.7)	0.020	5093	36 (0.7)	1	-

**Table 4:** Socio-demographic characteristics of first time blood donors at Fada N'gourma and Koudougou according to syphilis

	Fada N'gourma				Koudougou			
Characteristics	N Total	Syphilis Positive N (%)	OR (95% CI)	P-values	N Total	Syphilis Positive N (%)	OR (95% CI)	P-values
**Gender**								
Male	5001	84 (1.7)	1.8 (1.0-3.3)	0.034	3650	100 (2.7)	1.6 (0.9-2.6)	0.068
Female	1598	15 (0.9)	1	-	1177	21 (1.8)	1	-
**Age groups**								
< 20	1350	19 (1.4)	1.1 (0.4-3.2)	0.788	1093	27 (2.5)	1.6 (0.23-32.1)	0.534
20-29	3474	52 (1.5)	1.2 (0.5-3.1)	0.663	3031	79 (2.6)	1.7 (0.2-32.6)	0.511
30-39	1163	19 (1.6)	1.3 (0.5-3.7)	0.554	452	9 (2.0)	1.3 (0.2-27.0)	0.648
40-49	483	6 (1.2)	1	-	188	5 (2.7)	1.7 (0.2-39.1)	0.530
≥ 50	129	3 (2.3)	1.8 (0.4-8.6)	0.406	63	1 (1.6)	1	-
**Location of blood donation**								
Urban areas	4387	49 (1.1)	1	-	2801	69 (2.5)	1	-
Rural areas	2212	50 (2.3)	2.1 (1.4-3.1)	< 0.001	2026	52 (2.6)	1.0 (0.7-1.5)	0.821
**HBV**								
AgHBs positive	1198	23 (1.9)	1.4 (0.8-2.2)	0.186	705	18 (2.6)	1.0 (0.6-1.7)	0.932
AgHBs negative	5401	76 (1.4)	1	-	4122	103 (2.5)	1	-
**HCV**								
Seropositive	633	12 (1.9)	1.3 (0.7-2.5)	0.389	469	11 (2.3)	1	-
Seronegative	5699	87 (1.5)	1	-	4358	110 (2.5)	1.1 (0.6-2.1)	0.814
**HIV**								
Seropositive	147	2 (1.4)	1	-	141	3 (2.1)	1	-
Seronegative	6425	97 (1.5)	1.3 (0.3-7.8)	0.513	4686	118 (2.5)	1.2 (0.4-4.7)	0.526
**Blood groups**								
O	2908	42 (1.4)	1	-	2100	51 (2.4)	1	-
Non-O	3691	57 (1.5)	1.1 (0.7-1.6)	0.740	2727	70 (2.6)	1.1 (0.7-1.6	0.761

## Discussion

In the present report the seroprevalence of syphilis among first-time blood donors was 1.5%. This prevalence is relatively low compared to higher seroprevalence of 7.5% and 5.7% reported in previous studies respectively in Ghana [9] and Cameroon [10]. Syphilis' seroprevalence was higher in men compared to women. This finding could be explained by the high multiple sexual partners frequently observed in men compared to women [11]. The seroprevalence of syphilis showed a regional variation in blood donors in Burkina Faso in 2010; the highest was found in the Central-west region (Koudougou) and the lowest in the high-basins region (Bobo-Dioulasso). Syphilis' low prevalence has been previously reported in Bobo-Dioulasso [4]. However, the seroprevalence of 0.7% found in the present report is three times higher than the prevalence of 0.24% reported by Sombié et al (2000) in pregnant women.

We showed that syphilis in this study was not evenly distributed according to place of blood collection, as significantly higher seroprevalence was found in rural areas compared to urban ones among blood donors in Ouagadougou and Fada N′gourma. The low prevalence in urban areas could be explained by better prevention campaigns against sexually transmitted diseases compared to rural areas. The overall seroprevalence of syphilis was associated with HBV and HCV among blood donors. High co-infections of syphilis with HBV and HCV have been reported in previous studies [12, 13]. The seroprevalence of syphilis was associated with HBV and HCV among blood donors in Bobo-Dioulasso and Ouagadougou, respectively.

In many previous studies conducted in Africa [14, 15], it has been reported that sexually transmitted diseases such as syphilis increase the risk of HIV infection. Surprisingly, we did not find any association between HIV status and syphilis serology among blood donors. Our results are at variance with those of previous studies, but several arguments can be made to explain the observed discrepancy. Firstly, the lack of association between HIV and syphilis could indicate that the mode of transmission of both diseases may be partly distinct in first-time blood donors. Indeed, the high prevalence of syphilis among blood donors could be partly explained by the plausible existence of non-venereal Treponema in Burkina Faso. Indeed, eight countries have known endemic yaws in 2011 including four neighboring Burkina Faso such as Benin, Ivory Coast, Ghana and Togo [16]. Secondly, HIV and syphilis infections may not be spatially related in Burkina Faso. A recent study in South Africa has shown divergent spatial patterns in the prevalence of the HIV and syphilis in South African pregnant women. HIV was more prevalent in urban areas than elsewhere, while syphilis had a high prevalence in rural areas [17].

## Conclusion

We report on a representative sample of the population of Burkina Faso a low seroprevalence of syphilis. Syphilis' seroprevalence remains unequally distributed between urban and rural areas and was not associated with HIV infection.
